# Adults With Opioid and Methamphetamine Co-use Have Lower Odds of Completing Short-Term Residential Treatment Than Other Opioid Co-use Groups: A Retrospective Health Services Study

**DOI:** 10.3389/fpsyt.2021.784229

**Published:** 2021-12-08

**Authors:** Orrin D. Ware, Jennifer I. Manuel, Andrew S. Huhn

**Affiliations:** ^1^Behavioral Pharmacology Research Unit, Johns Hopkins University School of Medicine, Baltimore, MD, United States; ^2^Silver School of Social Work, New York University, New York, NY, United States

**Keywords:** co-use, short term treatment, methamphetamine, opioids, polysubstance use, treatment, residential, substance use disorder

## Abstract

**Objective:** There is an increase in persons entering substance use treatment who co-use opioids and methamphetamines in recent years. Co-using these substances may negatively impact treatment retention in the residential setting. We explored predictors of adults completing short-term residential treatment among persons with primary opioid use disorder (OUD) who co-use either alcohol, benzodiazepines, cocaine, or methamphetamines.

**Methods:** This study used the 2019 de-identified, publicly available Treatment Episode Dataset-Discharges. The sample included adults discharged from short-term residential treatment with primary OUD who co-used either alcohol, benzodiazepines, cocaine, or methamphetamines. The final sample size included 24,120 treatment episodes. Univariate statistics were used to describe the sample. Two logistic regression models were used to predict completing treatment. The first logistic regression model included the co-use groups as predictors and the second model added other demographic and treatment-relevant covariates.

**Results:** A slight majority (51.4%) of the sample prematurely discharged from treatment. Compared to the other three co-use groups, the opioid and methamphetamine co-use group had the highest proportion of individuals who were women (45.0%), unemployed (62.5%), current injection drug use (76.0%), living in the Midwest (35.9%), living in the south (33.5%), and living in the west (15.5%). The opioid and methamphetamine co-use group also had the highest proportion of individuals not receiving medications for OUD (84.9%), not having a prior treatment episode (28.7%), and not completing treatment (57.4%). In the final logistic regression model, which included covariates, the opioid and alcohol (OR = 1.18, 95% CI = 1.080–1.287, *p* < 0.001), opioid and benzodiazepine (OR = 1.33, 95% CI = 1.213–1.455, *p* < 0.001), and opioid and cocaine (OR = 1.16, 95% CI = 1.075–1.240, *p* < 0.001) co-use groups had higher odds of completing treatment than the opioid and methamphetamine co-use group.

**Conclusions:** Opioid and methamphetamine co-use may complicate short-term residential treatment retention. Future work should identify effective strategies to retain persons who co-use opioids and methamphetamines in treatment.

## Introduction

Different combinations of polysubstance use have been identified among individuals who use opioids, which must be considered in the context of opioid use disorder (OUD) treatment. One study among 356 people found over 55% of individuals with OUD co-use other substances such as alcohol, benzodiazepines, and cocaine (1). Sometimes substances are co-used to enhance or adjust for other drug effects (e.g., sedation, “comedown,” and withdrawal) (2–5). A recent trend in polysubstance use is the increase of opioid and methamphetamine co-use, which has been referred to as the fourth wave of the opioid crisis. Another epidemiological study in the United States identified a 66% increase in methamphetamine use from 2015 to 2018 among persons who used heroin in the past year, and a 49.2% increase among those with past twelve month prescription opioid misuse (6). Another epidemiological study found that past year use of both heroin and methamphetamine increased from 22.5% in 2015 to 46.7% in 2019 (7). Potential reasons for the increase of methamphetamine use include substituting the substance for opioids, a synergistic high, or balancing the effects of opioids (5).

Co-use of opioids and methamphetamine is associated with low socioeconomic status and health consequences such as overdose and increased need for medical care (8–10), and it is estimated that 10% of adults with OUD have a co-occurring methamphetamine use disorder (11). Compared to adults who use opioids alone, those with opioid and methamphetamine co-use were over 200% more likely to have housing instability, and had nearly 100% more hospital overnight stays and ~46% more visits to the emergency department (9). Regarding overdoses, data from 25 states show that methamphetamine was involved in over 10% of all opioid-related deaths (8). Along with the trends of opioid and methamphetamine co-use observed in the general population, this pattern was also identified among individuals entering treatment. One study found that adults admitted to treatment with opioid and methamphetamine co-use increased by ~10% from 1992 to 2017 (12). Further, individuals entering treatment with heroin as a primary substance had a 490% increase in methamphetamine co-use from 2008 to 2017 (13). Since polysubstance use is so prevalent in persons with OUD, understanding the complexities of co-using substances has the potential to enhance treatment (2, 14). Compared to those who discharge from treatment prematurely, longer retention or completing treatment is a predictor of better post-treatment outcomes. Some of these outcomes include increased harm reduction, longer periods of substance abstinence, and greater social functioning (15, 16).

Residential treatment is recommended for individuals who are unstable and have moderate to severe substance use disorders (15, 17). While longer stays in treatment (e.g., >90 days) are associated with positive outcomes for opioids, methamphetamines, and other drug use generally (16, 18–21), the benefit of short-term residential treatment (e.g., 30 days or less) on the co-use of opioids and methamphetamines is unclear. Co-use of opioids and methamphetamines has the potential to impact aspects of treatment such as behavioral counseling and withdrawal management. Although medications for OUD (MOUD) are effective in improving relapse and retention outcomes, individuals who co-use substances with opioids are less likely to receive these medications (12, 22). Persons with OUD are less likely to receive MOUD in short-term residential compared with outpatient treatment (23), and there is also a large gap between availability of MOUD and use of MOUD in residential treatment facilities across the U.S (24). Treatment for co-occurring opioid and methamphetamine use disorders is further complicated by the lack of effective pharmacotherapy options for methamphetamine use (25–27).

This study examined differences in demographic, drug use, and treatment characteristics among adults discharged from short-term residential treatment in four distinct opioid co-use groups: (1) alcohol, (2) benzodiazepines, (3) cocaine, and (4) methamphetamine. These four groups were chosen because another study that examined these groups found a decrease in the prevalence of opioid and alcohol co-use and opioid and cocaine co-use, and an increase in the prevalence of opioid and benzodiazepine co-use and opioid and methamphetamine co-use (12). Further, this study examined treatment completion rates from short-term residential treatment for persons with OUD as a function of co-use drug classes.

## Materials and Methods

### Sample

The publicly available de-identified Treatment Episode Dataset–Discharges (TEDS-D) 2019, which is provided annually by the Substance Abuse and Mental Health Services Administration was used for this study (28). TEDS-D contains demographic information, substance use characteristics, treatment type, and discharge information for treatment episode discharges in the year of 2019 from U.S. substance use treatment providers that receive public funds (28). States that were excluded from the dataset due to insufficient data include Oregon, Washington, and West Virginia (28). The sample was selected by using the following criteria: (1) 18 years or older, (2) admitted to short-term residential treatment, (3) discharged from short-term residential treatment, (4) has a value for the outcome, reason for discharge, (5) death was not the reason for discharge, (6) heroin, non-prescription methadone, or other opiates and synthetics was the primary substance, and (7) alcohol, benzodiazepines, cocaine, or methamphetamine/speed was the secondary substance.

### Measures

Co-use groups were created if opioids were the primary substance and alcohol, benzodiazepines, cocaine, and methamphetamine/speed were the secondary substances. The discharge reason was dichotomized as treatment completed and premature discharge. Length of stay was a continuous variable with values from 1 to 30 which describes the length of the treatment episode in days. Age was recoded to include the following age ranges 18–29 years old, 30–39 years old, 40–49 years old, and 50 years and older. Race was recoded with the following categories, Black, White, and Other. Non-Black and non-White groups were combined into the Other category due to low frequencies. Gender was a binary variable with women and men as values. Receiving medication for opioid use disorder (MOUD) in the current treatment plan was dichotomized as Yes or No. The frequency of use variables (primary substance and secondary substance) had No use, Some use, and Daily use as values. Prior substance use treatment refers to ever having a previous substance use treatment episode and was dichotomized as Yes or No.

### Analyses

Univariate analyses including counts, percentages, and means were used to describe the full sample and the four co-use groups. An analysis of variance (ANOVA) was used to examine the associations between the co-use groups and length of stay in treatment. Two logistic regression models were conducted to predict completing treatment. Little's Missing Completely at Random (MCAR) test was utilized for treatment episodes missing values for variables included in the final logistic regression model. Little's MCAR test (*p* = 0.134) provided evidence that listwise deletion was adequate. Listwise deletion was used for treatment episodes that were missing values for variables included in the final logistic regression model. The first logistic regression model included only the co-use groups as predictors (reference group: Opioid and Methamphetamine co-use group). The second logistic regression model retained the co-use groups and added the following covariates: age (reference group: 18–29 years old), gender (reference group: Women), race (reference group: White), receiving MOUD (reference group: No), frequency of primary substance (reference group: Daily), frequency of secondary substance (reference group: Daily), and prior substance use treatment (reference group: No). Due to the large sample size, *p* < 0.001 was established as the threshold for significance in the bivariate and multivariate analyses. Analyses were performed using SPSS Version 27 (Armonk, NY).

## Results

### Sample Characteristics

There were 28,483 treatment episodes that met initial eligibility criteria, however 4,363 did not have complete data and were thus excluded, leaving a final sample of *n* = 24,120 treatment episodes. There were 3,918 (16.2%) treatment episodes in the opioids and alcohol co-use group, 3,230 (13.4%) in the opioids and benzodiazepines co-use group, 11,575 (48.0%) in the opioids and cocaine co-use group, and 5,397 (22.4%) in the opioids and methamphetamine co-use group.

Demographic, substance use, and treatment characteristics of the full sample and the four co-use groups are shown in Table 1. Less than half of the full sample completed treatment (48.6%). Most of the full sample were men (63.6%), White (72.9%), and not Hispanic or Latino (86.7%). Proportionally, the opioid and methamphetamine co-use group had the largest percentage in the Midwest (35.9%), South (33.5%), and West (15.5%) regions. Similarly, the opioid and methamphetamine co-use group had the largest combined proportion of individuals ages 18–39 years old (84.5%), followed by the opioid and benzodiazepine co-use group (79.4%). Women were the largest percent in the opioid and methamphetamine co-use group (45.0%) followed by the opioid and benzodiazepine co-use group (36.4%). The opioid and methamphetamine co-use group also had a higher percentage of cases being unemployed (62.5%), engaging in injection drug use (76.0%), not currently receiving MOUD (28.7%), and not having a prior substance use treatment episode (28.7%). Regarding days in treatment, the opioid and methamphetamine co-use group had an average of 18.4 (SD = 10.8) days, opioid and alcohol co-use group had an average of 16.2 (SD = 9.8) days, opioid and cocaine co-use group had an average of 15.9 (SD = 9.8) days, and the opioid and benzodiazepine group had an average of 15.2 (SD = 9.6). Results from an analysis of variance indicated the co-use groups influenced the number of days in treatment [*F*_(3, 24, 116)_ = 95.97, *p* < 0.001]. Using Tukey's honest significant difference, the opioid and methamphetamine group had a significantly higher average number of days in treatment than the other three co-use groups.

**Table 1:** Demographic, substance use, and treatment characteristics of the full sample and co-use groups.

	**Full study sample (*n*, %)**	**Opioids and alcohol subsample (*n*, %)**	**Opioids and benzodiazepines subsample (*n*, %)**	**Opioids and cocaine subsample (*n*, %)**	**Opioids and methamphetamines subsample (*n*, %)**
Sample size	24,120 (100.0%)	3,918 (100.0%)	3,230 (100.0%)	11,575 (100.0%)	5,397 (100.0%)
Days in treatment, mean (Standard deviation)	16.4 (SD = 10.1)	16.2 (SD = 9.8)	15.2 (SD = 9.6)	15.9 (SD = 9.8)	18.4 (SD = 10.8)
**Region**					
Northeast	11,547 (47.9%)	2,071 (52.9%)	1,789 (55.4%)	6,871 (59.4%)	816 (15.1%)
Midwest	6,570 (27.2%)	1,012 (25.8%)	677 (21.0%)	2,946 (25.5%)	2,935 (35.9%)
South	4,896 (20.3%)	732 (18.7%)	707 (21.9%)	1,649 (14.2%)	1,808 (33.5%)
West	1,107 (4.6%)	103 (2.6%)	57 (1.8%)	109 (0.9%)	838 (15.5%)
**Age**					
18–29 years old	7,704 (31.9%)	946 (24.1%)	1,370 (42.4%)	3,104 (26.8%)	2,284 (42.3%)
30–39 years old	9,024 (37.4%)	1,254 (32.0%)	1,194 (37.0%)	4,300 (37.1%)	2,276 (42.2%)
40–49 years old	4,023 (16.7%)	737 (18.8%)	397 (12.3%)	2,215 (19.1%)	674 (12.5%)
50 years and older	3,369 (14.0%)	981 (25.0%)	269 (8.3%)	1,956 (16.9%)	163 (3.0%)
**Education level^a^**					
Less than HS Diploma or GED	6,047 (25.1%)	973 (24.8%)	605 (18.7%)	3,132 (27.1%)	1,337 (24.8%)
HS Diploma or GED	11,585 (48.0%)	1,873 (47.8%)	1,581 (48.9%)	5,494 (47.5%)	2,637 (48.9%)
1–3 years of college, university, vocational	5,187 (21.5%)	844 (21.5%)	796 (24.6%)	2,334 (20.2%)	1,213 (22.5%)
4 years of college, university or higher	1,151 (4.8%)	212 (5.4%)	224 (6.9%)	548 (4.7%)	167 (3.1%)
Missing	150 (0.6%)	16 (0.4%)	24 (0.7%)	67 (0.6%)	43 (0.8%)
**Gender**					
Women	8,782 (36.4%)	999 (25.5%)	1,177 (36.4%)	4,175 (36.1%)	2,431 (45.0%)
Men	15,338 (63.6%)	2,919 (74.5%)	2,053 (63.6%)	7,400 (63.9%)	2,966 (55.0%)
**Race**					
Black	3,915 (16.2%)	880 (22.5%)	240 (7.4%)	2,636 (22.8%)	159 (2.9%)
White	17,581 (72.9%)	2,555 (65.2%)	2,736 (84.7%)	7,393 (63.9%)	4,897 (90.7%)
Other	2,624 (10.9%)	483 (12.3%)	254 (7.9%)	1,546 (13.4%)	341 (6.3%)
**Ethnicity**					
Hispanic or Latino	3,011 (12.5%)	531 (13.6%)	286 (8.9%)	1,752 (15.1%)	442 (8.2%)
Not Hispanic or Latino	20,913 (86.7%)	3,346 (85.4%)	2,916 (90.3%)	9,736 (84.1%)	4,915 (91.1%)
Missing	196 (0.8%)	41 (1.0%)	28 (0.9%)	87 (0.8%)	40 (0.7%)
**Employment status**					
Full-time	1,620 (6.7%)	341 (8.7%)	336 (10.4%)	649 (5.6%)	294 (5.4%)
Part-time	568 (2.4%)	107 (2.7%)	102 (3.2%)	271 (2.3%)	88 (1.6%)
Unemployed	9,826 (40.7%)	1,358 (34.7%)	1,096 (33.9%)	3,997 (34.5%)	3,375 (62.5%)
Not in labor force	11,944 (49.5%)	2,091 (53.4%)	1,680 (52.0%)	6,585 (56.9%)	1,588 (29.4%)
Missing	162 (0.7%)	21 (0.5%)	16 (0.5%)	73 (0.6%)	52 (1.0%)
**Housing status**					
Homeless	7,356 (30.5%)	1,246 (31.8%)	695 (21.5%)	3,948 (34.1%)	1,467 (27.2%)
Dependent living	4,243 (17.6%)	639 (16.3%)	515 (15.9%)	1,747 (15.1%)	1,342 (24.9%)
Independent living	12,280 (50.9%)	2,013 (51.4%)	1,990 (61.6%)	5,787 (50.0%)	2,490 (46.1%)
Missing	241 (1.0%)	20 (0.5%)	30 (0.9%)	93 (0.8%)	98 (1.8%)
**Age first used primary substance**					
11 years and under	363 (1.5%)	62 (1.6%)	43 (1.3%)	126 (1.1%)	132 (2.4%)
12–14 years old	1,767 (7.3%)	305 (7.8%)	209 (6.5%)	683 (5.9%)	570 (10.6%)
15–17 years old	3,799 (15.8%)	600 (15.3%)	574 (17.8%)	1,704 (14.7%)	921 (17.1%)
18–20 years old	5,030 (20.9%)	782 (20.0%)	745 (23.1%)	2,396 (20.7%)	1,107 (20.5%)
21–24 years old	4,423 (18.3%)	611 (15.6%)	677 (21.0%)	2,170 (18.7%)	965 (17.9%)
25–29 years old	3,890 (16.1%)	616 (15.7%)	477 (14.8%)	1,971 (17.0%)	826 (15.3%)
30 years and older	4,734 (19.6%)	922 (23.5%)	495 (15.3%)	2,469 (21.3%)	848 (15.7%)
Missing	114 (0.5%)	20 (0.5%)	10 (0.3%)	56 (0.5%)	28 (0.5%)
**Frequency of primary substance use**					
No use in the past month	2,851 (11.8%)	396 (10.1%)	252 (7.8%)	1,025 (8.9%)	1,178 (21.8%)
Some use	4,569 (18.9%)	703 (17.9%)	457 (14.1%)	1,899 (16.4%)	1,510 (28.0%)
Daily use	16,700 (69.2%)	2,819 (71.9%)	2,521 (78.0%)	8,651 (74.7%)	2,709 (50.2%)
**Age first used secondary substance**					
11 years and under	751 (3.1%)	420 (10.7%)	43 (1.3%)	125 (1.1%)	143 (2.6%)
12–14 years old	2,811 (11.7%)	1,195 (30.5%)	209 (6.5%)	756 (6.5%)	563 (10.4%)
15–17 years old	4,888 (20.3%)	1,149 (29.3%)	574 (17.8%)	2,137 (18.5%)	877 (16.2%)
18–20 years old	4,456 (18.5%)	463 (11.8%)	745 (23.1%)	2,469 (21.3%)	904 (16.8%)
21–24 years old	3,133 (13.0%)	207 (5.3%)	677 (21.0%)	1,728 (14.9%)	801 (14.8%)
25–29 years old	2,905 (12.0%)	81 (2.1%)	477 (14.8%)	1,565 (13.5%)	837 (15.5%)
30 years and older	3,249 (13.5%)	124 (3.2%)	495 (15.3%)	1,603 (13.8%)	960 (17.8%)
Missing	1,927 (8.0%)	279 (7.1%)	10 (0.3%)	1,192 (10.3%)	312 (5.8%)
**Frequency of secondary substance use**					
No use in the past month	2,949 (12.2%)	423 (10.8%)	307 (9.5%)	1,060 (9.2%)	1,159 (21.5%)
Some use	8,618 (35.7%)	1,226 (31.3%)	1,001 (31.0%)	4,124 (35.6%)	2,267 (42.0%)
Daily use	12,553 (52.0%)	2,269 (57.9%)	1,922 (59.5%)	6,391 (55.2%)	1,971 (36.5%)
**Current injection drug use**					
Yes	14,526 (60.2%)	2,141 (54.6%)	1,740 (53.9%)	6,907 (59.7%)	4,102 (76.0%)
No	9,594 (39.8%)	1,777 (45.4%)	1,490 (46.1%)	4,668 (40.3%)	1,295 (24.0%)
**Receiving medication for opioid use disorder**					
Yes	6,504 (27.0%)	946 (24.1%)	922 (28.5%)	3,821 (33.0%)	815 (15.1%)
No	17,616 (73.0%)	2,972 (75.9%)	2,308 (71.5%)	7,754 (67.0%)	4,582 (84.9%)
**Prior substance use treatment**					
Yes	20,321 (84.2%)	3,377 (86.2%)	2,732 (84.6%)	10,363 (89.5%)	3,849 (71.3%)
No	3,799 (15.8%)	541 (13.8%)	498 (15.4%)	1,212 (10.5%)	1,548 (28.7%)
**Discharge reason**					
Treatment completed	11,719 (48.6%)	1,978 (50.5%)	1,677 (51.9%)	5,764 (49.8%)	2,300 (42.6%)
Premature discharge	12,401 (51.4%)	1,940 (49.5%)	1,553 (48.1%)	5,811 (50.2%)	3,097 (57.4%)

*Some percents may not equal to 100% due to rounding error. Percents are column percents*.

a *HS, High School; GED, General Educational Development*.

### Predicting Treatment Completion

Results from the first logistic regression model are shown in Table 2. In model 1, which excluded covariates, the opioid and alcohol (OR = 1.37, 95% CI = 1.264–1.491, *p* = < 0.001), opioid and benzodiazepine (OR = 1.454, 95% CI = 1.332–1.587, *p* < 0.001), and opioid and cocaine (OR = 1.33, 95% CI = 1.251–1.425, *p* < 0.001) co-use groups all had higher odds of completing treatment than the opioid and methamphetamine co-use group.

**Table 2:** Logistic regression model predicting treatment completion by co-use groups.

**Variable**	**Odds ratio**	**95% CI**	***p***
**Co-use groups (Ref:Opioid+Methamphetamine)**			
Opioid+Alcohol	1.373	1.264–1.491	<0.001
Opioid+Benzodiazepine	1.454	1.332–1.587	<0.001
Opioid+Cocaine/Crack	1.336	1.251–1.425	<0.001

Results from the final logistic regression model are shown in Table 3. In model 2, which included covariates, the opioid and alcohol (AOR = 1.18, 95% CI = 1.080–1.287, *p* < 0.001), opioid and benzodiazepine (AOR = 1.33, 95% CI = 1.213–1.455, *p* < 0.001), and opioid and cocaine (AOR = 1.16, 95% CI = 1.075–1.240, *p* < 0.001) co-use groups also had higher odds of completing treatment than the opioid and methamphetamine co-use group. Individuals aged ≥50 years old (AOR = 1.40, 95% CI = 1.278–1.538, *p* < 0.001) had higher odds of completing treatment than those between the ages of 18–29 years old. Men (AOR = 1.26, 95% CI = 1.190–1.326, *p* < 0.001) had higher odds of completing treatment than women. Those who were Black had lower odds (AOR = 0.85, 95% CI = 0.784–0.921, *p* < 0.001) of completing treatment than those who were White. Conversely, those whose race was categorized as “Other” (AOR = 1.34, 95% CI = 1.233–1.464, *p* < 0.001) had higher odds of completing treatment than those who were White. Treatment episodes that received MOUD (AOR = 1.63, 95% CI = 1.537–1.731, *p* < 0.001) had higher odds of completing treatment than those that did not. Individuals who used their primary opioid substance sometimes (AOR = 1.15, 95% CI = 1.065–1.238, *p* < 0.001) had higher odds of completing treatment than those who used their primary substance daily. Conversely, individuals who used their secondary substance sometimes (AOR = 0.90, 95% CI = 0.843–0.955, *p* < 0.001) had lower odds of completing treatment than those who used their secondary substance daily.

**Table 3:** Logistic regression model predicting treatment completion by co-use groups and covariates.

**Variable**	**Adjusted odds ratio**	**95% CI**	***p***
**Co-use groups (Ref:Opioid+Methamphetamine)**			
Opioid+Alcohol	1.18	1.080–1.287	<0.001
Opioid+Benzodiazepine	1.33	1.213–1.455	<0.001
Opioid+Cocaine/Crack	1.16	1.075–1.240	<0.001
**Age groups (Ref: 18–29 years old)**			
30–39 years old	1.02	0.959–1.085	0.528
40–49 years old	1.07	0.989–1.159	0.093
50 years and older	1.402	1.278–1.538	<0.001
**Gender (Ref: Women)**			
Men	1.26	1.190–1.326	<0.001
**Race (Ref: White)**			
Black	0.85	0.784–0.921	<0.001
Other	1.34	1.233–1.464	<0.001
**Receiving medication for opioid use disorder (Ref: No)**			
Yes	1.63	1.537–1.731	<0.001
**Frequency of use primary substance (Ref: Daily)**			
Some use	1.15	1.065–1.238	<0.001
No use	1.19	1.053–1.341	0.005
**Frequency of use secondary substance (Ref: Daily)**			
Some use	0.90	0.843–0.955	<0.001
No use	0.89	0.784–0.999	0.048
Prior substance use treatment (Ref: No)
Yes	1.11	1.036–1.197	0.004

*Due to the large sample size p < 0.001 was established as the threshold for significance*.

## Discussion

The current study identified adults who co-use opioids and methamphetamine as having lower odds of completing treatment than other opioid co-use groups, namely alcohol, benzodiazepines, and cocaine. Considering the alarming increase of opioid and methamphetamine co-use in recent years (6, 7), this group's heightened risk of treatment attrition requires attention by treatment providers and researchers. This study also found the opioid and methamphetamine co-use group had significantly more days in treatment than other co-use groups. It is interesting that this group had the highest proportion of not completing treatment yet had the longest number of days in treatment. Perhaps this points to treatment providers considering a longer course of treatment necessary to adequately treat individuals who co-use opioids and methamphetamines.

This study also found that the opioid and methamphetamine co-use group had a higher proportion of women than the other three co-use groups. A review of the literature found that women start using methamphetamine at an earlier age and are more dependent on methamphetamine than men (29). Among reproductive age and pregnant women, methamphetamine is one of the most abused substances (30, 31). A study based on persons who inject drugs in Seattle found a higher proportion of women co-using heroin and amphetamine instead of using these substances alone (32). Women with OUD have also been found to have higher rates of treatment attrition in multiple studies (33).

The regional distribution of opioid and methamphetamine co-use is also noteworthy. This co-use group had the highest proportion in the Southern, Midwestern, and Western regions. While data have shown that methamphetamine use and related overdose deaths are more common in the Western region (34), recent data show that methamphetamine use is expanding to other geographic areas in Southern and Midwestern regions (35, 36), which are already epicenters of the opioid crisis.

The opioid and methamphetamine co-use group had the highest proportion of injection drug use, as over three-fourths of the treatment episodes in this group indicated current injection drug use. Another study found that opioid and methamphetamine co-use was associated with a 132% higher prevalence of injection drug use when compared to those who only use opioids (37). Injection drug use is associated more severe substance use disorder, which itself increases the risk of premature discharge from treatment. Not receiving MOUD increases the risk of treatment attrition among persons with OUD (12). The opioid and methamphetamine co-use group had the lowest proportion of receiving MOUD in this study. Although effective medications to treat methamphetamine are lacking (25–27), the medications to treat OUD may increase treatment completion in this co-use group.

This study is not without limitations. One limitation is this study focused on treatment episodes from treatment providers that receive public funding. These results may not be generalizable to private substance use treatment providers. Including treatment episodes that do not use their primary or secondary substance in the past month is a limitation, although this subgroup might be fundamentally different than those who enter treatment with active use. A second limitation is only including primary and secondary substances while excluding tertiary substances. Although data were analyzed in this way to focus on the two main substances, if tertiary substances were considered there could be potential overlap between the co-use groups. For example, an individual could use heroin, methamphetamine, and cocaine prior to entering short-term residential treatment. This creates a challenge for group comparisons as there are several potential 3-group combinations of polysubstance use, and it is difficult to interpret the importance of tertiary drug use within the TEDS dataset. A third limitation is the potential duplication or overestimation of polysubstance use given that TEDS-D cases are discharges and not individuals, although we countered this limitation by controlling for prior treatment episodes in the multivariate model. Since this study utilized a secondary dataset, we were constrained by the available variables. Considering this limitation, we were unable to include other predictors such as sexual orientation, type of medication for OUD, family support, and treatment provider characteristics. Finally, we were limited by not having follow-up data beyond discharge from treatment, and although completing treatment is associated with better posttreatment outcomes, this cannot be assessed in the current study. Considering these limitations, this study provides key insight into opioid co-use groups and short-term residential treatment completion.

## Data Availability Statement

Publicly available datasets were analyzed in this study. This data can be found at: https://www.datafiles.samhsa.gov/dataset/teds-d-2019-ds0001-teds-d-2019-ds0001.

## Author Contributions

All authors have contributed to the design, preparation, and editing of the manuscript.

## Funding

This research was funded by the National Institute on Drug Abuse (NIDA) T32 training grant (T32DA007209, Bigelow/Strain/Weerts).

## Conflict of Interest

AH receives research funding from Ashley Addiction Treatment through his university. The remaining authors declare that the research was conducted in the absence of any commercial or financial relationships that could be construed as a potential conflict of interest. The reviewer PM declared a shared affiliation, with no collaboration, with the author JM at the time of the review.

## Publisher's Note

All claims expressed in this article are solely those of the authors and do not necessarily represent those of their affiliated organizations, or those of the publisher, the editors and the reviewers. Any product that may be evaluated in this article, or claim that may be made by its manufacturer, is not guaranteed or endorsed by the publisher.
